# Development of Fast E-nose System for Early-Stage Diagnosis of Aphid-Stressed Tomato Plants

**DOI:** 10.3390/s19163480

**Published:** 2019-08-09

**Authors:** Shaoqing Cui, Elvia Adriana Alfaro Inocente, Nuris Acosta, Harold. M. Keener, Heping Zhu, Peter P. Ling

**Affiliations:** 1 Department of Food, Agricultural and Biological Engineering, The Ohio State University/Ohio Agricultural Research and Development Center, 1680 Madison Ave, Wooster, OH 44691-4096, USA; 2 Department of Entomology, The Ohio State University/Ohio Agricultural Research and Development Center, 1680 Madison Ave, Wooster, OH 44691-4096, USA; 3 United States Department of Agriculture-Agricultural Research Service (USDA-ARS) Application Technology Research Unit, 1680 Madison Ave, Wooster, OH 44691-4096, USA

**Keywords:** aphid, electronic nose, sensors, tomato plant, volatile organic compounds (VOCs), biomarkers

## Abstract

An electronic nose (E-nose) system equipped with a sensitive sensor array was developed for fast diagnosis of aphid infestation on greenhouse tomato plants at early stages. Volatile organic compounds (VOCs) emitted by tomato plants with and without aphid attacks were detected using both the developed E-nose system and gas chromatography mass spectrometry (GC-MS), respectively. Sensor performance, with fast sensor responses and high sensitivity, were observed using the E-nose system. A principle component analysis (PCA) indicated accurate diagnosis of aphid-stressed plants compared to healthy ones, with the first two PCs accounting for 86.7% of the classification. The changes in VOCs profiles of the healthy and infested tomato plants were quantitatively determined by GC-MS. Results indicated that a group of new VOCs biomarkers (linalool, carveol, and nonane (2,2,4,4,6,8,8-heptamethyl-)) played a role in providing information on the infestation on the tomato plants. More importantly, the variation in the concentration of sesquiterpene VOCs (e.g., caryophyllene) and new terpene alcohol compounds was closely associated with the sensor responses during E-nose testing, which verified the reliability and accuracy of the developed E-nose system. Tomato plants growing in spring had similar VOCs profiles as those of winter plants, except several terpenes released from spring plants that had a slightly higher intensity.

## 1. Introduction

Plant diseases and pests infestation have resulted in large economic losses in agriculture production. For example, one trail estimated that tomato yield loss resulting from insect damage was around 30% [1]. Early diagnosis of infested plants prior to the onset of visual symptoms is very valuable for executing appropriate management strategies and pest control to prevent the spread of diseases [2,3]. However, it is difficult to determine the physical, chemical, and biological characteristics of infested plants during the asymptomatic stages, in which the infestation features were not obvious. Another challenge lies in the difficulty of detecting infestations in a timely and economic manner. 

Many methods and procedures have been developed to address these challenges, and new technologies are continuously emerging. The most widely used technologies for the diagnosis of plant pests are laboratory-based methods, such as polymerase chain reaction (PCR) [4,5,6,7]. However, these methods are time-consuming, expensive, and destructive. A more recent technology is the detection of Volatile organic compounds (VOCs) released from plants, which has received extensive attention due to its noninvasive and rapid features [8]. It has been known that VOCs play significant roles in plant communication, plant defense in combating infections, and attacks by herbivorous insects; thus, they show promise for improving plant protection [9]. In other words, the VOCs profiles, known as VOCs fingerprints, released from plants in different stages indicate their real-timely physiological status, and can be used as bio-information for insect diagnosis at early stages. 

A conventional gas chromatography–mass spectrometry (GC-MS) is widely used for determining each VOC for plant status screening [10,11]. Borges et al. used GC-MS to compare the difference in volatile compounds emitted by passion vine plants (*Passiflora edulis*) injured by three types of damage (mechanical damage, herbivory, and oviposition (*Lepidopteran: Helicontius erato*) infestation) [11,12]. However, GC-MS is expensive and time-consuming, and requires laboratory-based testing that limits its application for real-time testing in greenhouses and fields. To fully take advantage of plant VOC information for insect management, portable, easy to operate, real-time detection technology is required. An artificial intelligent nose or Electronic nose (E-nose), which is designed to mimic the functionality of a human nose, provides a rapid and real-time approach to detect VOCs. It is coupled with different types of sensor arrays, a signal conditioning module, a data acquisition unit, and pattern recognition algorithms that emulate the roles of the olfactory bulb, nervous system, and brain [13]. Such technology has been widely developed in the past two decades and extensively employed in diverse applications ranging from medical diagnosis to the food industry, environmental protection, and agriculture [14,15,16]. 

Specifically, diagnosis for plant insects and diseases before and after harvest by using E-nose system has attracted much attention due to its fast, noninvasive, and reliable approach. The E-nose system has been used for the detection of both the duration and prevalence of red flour beetle infestation in stored rough rice [17]. Different fungi species can be correctly classified by using the E-nose system based on their odor profiles [18]. The fungal colony counts in peaches in different storage times were successfully predicted by using the E-nose system, demonstrating the potential use of E-nose to discriminate among fungal contamination in peaches [19]. Infested plants attacked by the striped rice stem borer and the brown plant hopper were reported by determining the changes in the VOCs fingerprint by using an E-nose system. Besides testing plant VOCs, mating disruption, which is used to confuse males to impede mating, thus reducing the amount of insects, is widely adopted. A portable E-nose was used to predict the gender and species of stink bugs prior to applying mating disruption methods [20,21]. With the discoveries of biomarker metabolites and aroma profiles, the use of E-nose has been extended to plant insect control and disease diagnosis through the approaches of VOCs closely associated with individual diseases. Specific for insect control, the E-nose system and solid-phase micro-extraction (SPEM) extraction technique has been used for a rapid detection and classification of Hendel infestation in citrus fruits, insect attacks on tea plants, mechanical damages in tea plants, and *Rhyzopertha dominica* infestation in wheat [22,23,24,25,26]. Several biomarkers, such as 2-hepanol, 2-methylpropanoic acid and 2,3,5-trimethylpyrazine were used for the classification of quality and the origins of cocoa beans through the analytical approaches of E-nose and GC-MS with SPME extraction technique [27]. VOCs biomarkers, such as methyl salicylate and terpenes, were reported closely related to whitefly-infested plants [28]. However, development of a reliable E-nose system specifically for diagnosis of insect damage in greenhouse is still under development. 

The objective of this research was to design and develop an E-nose system with a sensitive sensor array and Labview interface for early-stage diagnosis of aphid-infested tomato plants in a greenhouse. The goal was to detect plant VOCs for the purpose of accurately and quickly distinguishing between healthy tomato plants and aphid-stressed ones at the beginning of infestation. 

## 2. Methods and Materials

### 2.1. Experiment Design 

**Figure 1:**
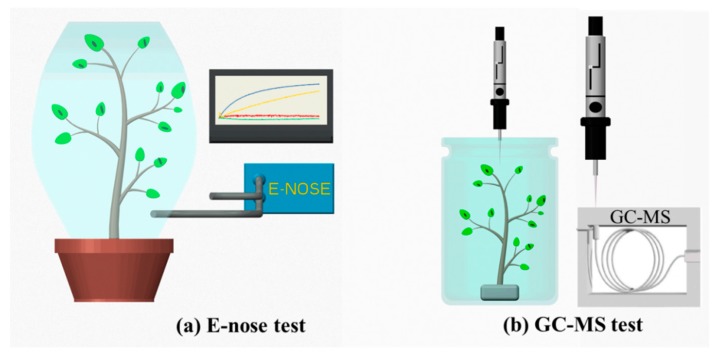
Illustration of early diagnosis of aphids-stressed tomato plants by using E-nose and GC-MS.

The research was carried out in three steps. First, an E-nose system was developed and optimized (details see Section 2.2) in the lab. Second, tomato plants were cultivated in a greenhouse for six weeks. Starting from the seventh week, the plants were cultivated under two different treatments: (1) induced aphid (*Myzus persicae*) infestation for two days, and (2) no exposure to aphids (control group). The last step was to determine VOCs of tomato plants using the E-nose test and GC-MS test, separately, as illustrated in Figure 1. As shown, tomato plants (with and without aphid infestation) were sealed in Teflon bags to generate headspace gas for E-nose tests (Figure 1a). Then, samples from the same tomato plant were sealed in a clean glass bottle for GC-MS analysis via solid-phase micro-extraction (SPME) absorbing VOCs (Figure 1b). The fiber material of the SPME was Carboxen/PDMS. E-nose and GC-MS tests are described further in Section 2.3 and Section 2.4, respectively. 

### 2.2. Fabrication of E-nose System

**Figure 2:**
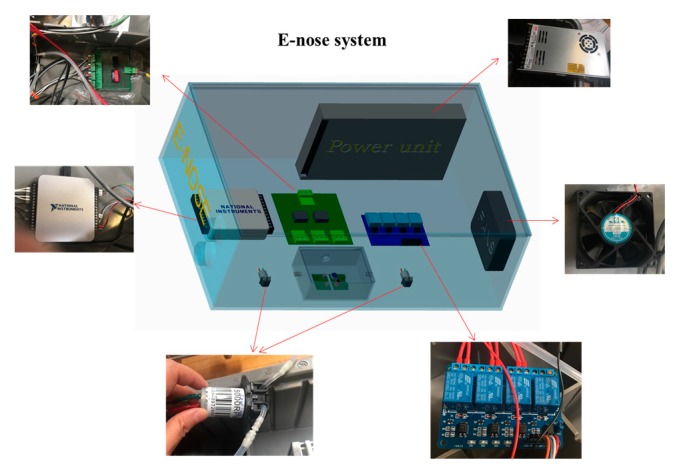
Illustration of E-nose system.

A portable E-nose system equipped with a sensor, including four different gas sensors array, a gas chamber with the sensor array sealed inside, a signal conditioning circuit, data acquisition, a fan for temperature control, and two gas pumps for pumping sample gases and pumping clean gas was developed in our intelligent sprayer workshop (United State Department of Agriculture–Application Technology Research, Wooster, OH, USA) and plant sensing lab (Ohio State University, Wooster, OH, USA). The property of each sensor is listed in Table 1. Specifically, the reasons for choosing MQ 138 and TGS 2602 were due to their sensitivity to VOCs and aromatic gases. And reasons for MQ 135 and MQ 3 were because of their sensitivity to alcohols and benzene, which were reported to be potential bio-markers of infested plants. All hardware components were integrated into a plastic box (Bud Industries Inc., Willoughby, IL, USA). Two micro pumps (Model NMS020L, KNF Neuberger Inc., Trenton, NJ, USA) were connected to a sealed gas chamber through polyethylene tubes. The sensor array was sealed into the gas chamber. Polyethylene tubes were used to connect two pumps and two valves (Nalgene Stopcock with 2 mm Bore, Thermo Scientific, Chardon, OH). A multiple power supply (RT-50 A, Mean Well USA, Inc. Fremont, CA, USA) was employed to supply various voltages, and the developed E-nose system required a voltage of 12 V DC. A data acquisition device (USB6200, National Instrumental Inc. Austin, TX, USA) was chosen to control the pumps and valves and to measure the voltage of each sensor. The interface software, based on the Labview platform (National Instrumental Inc. Austin, TX, USA), was used to control the whole system and display real-time sensor responses. The volatile value along with the detection time of each sensor was automatically saved in TXT format. A picture of the developed E-nose system is illustrated in Figure 2. 

### 2.3. E-nose Data Collection 

Tomato plants with different treatments (with and without aphid attacks) were sealed in Teflon bags for 3 hours to generate headspace gases, as shown in Figure 1a. Before testing, the E-nose system was rinsed with nitrogen for 5 min to reach its reference value. Subsequently, the produced headspace gas was pumped into the E-nose system for another 5 min through a needle connected to the sealed Teflon bag. The operational parameters are listed in Table 2. Ten tomato plant samples of each treatment for each treatment were tested (10 duplicates × 2 treatments). Principle component analysis (PCA) based on maximum values of each sensor response was used to analyze the data. 

### 2.4. GC-MS Detection

For detection using GC-MS, a 12~13 cm branch was taken from each tomato plant tested by the E-nose system, and each was sealed in a clean glass bottle (20 cm height) for 60 min at the room temperature to produce headspace gases, as shown in Figure 1b. The aphid-stressed tomato plant samples were prepared by placing 35 aphids on each tomato plant with each leaf having 7~8 aphids. The tomato plant was cultivated for 3 days before being sealed for 60 min. Each sample was replicated 3 times. The headspace gases were absorbed by Solid-Phase Micro Extraction (SPME), and the collected VOCs were desorbed, separated through the column (ZB-5HT, 30 m × 0.25 mm × 0.25 mm, Zebron, Phenomenex, CA, USA), and then identified by MS in the GC-MS system (QP 2010 SE, Shmadzu, Kyoto, Japan). 

The GC-MS operational parameters included a splitless injection carried out at 200 °C for 3 min for desorption, and an injected volume of 2 μL. Following the injection, the column temperature was programmed from 50 °C (1 min) to 120°C at 5 °C per min, increased to 240 °C at 8 °C per min, and kept for 5 mins. The ion source was set at 230 °C. The mass-spectra was at 70 eV within a mass range of 40 to 350 amu. Compounds were identified by comparing the recorded mass spectra with the National Institute of Standards and Technology mass spectral library, retention index (RI), and previous literature and published index data (www.flavornet.org) [22,23,24,25,26,27]. The retention indices were calculated from all of the volatile compounds using a homologous series of n-alkanes (C8 to C20) (Sigma-Aldrich Co., Ltd.). 

## 3. Results and Discussion

### 3.1. E-nose Sensor Response and Data Analysis

**Figure 3:**
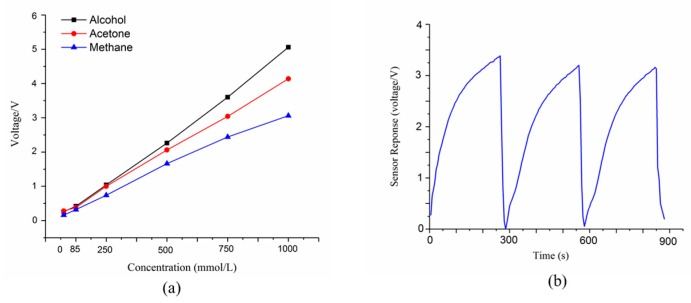
Sensor training of sensor MQ 138 towards pure gases with different concentrations (**a**), and the reproducibility test of sensor MQ 138 under exposure to healthy tomato plant (**b**).

**Figure 4:**
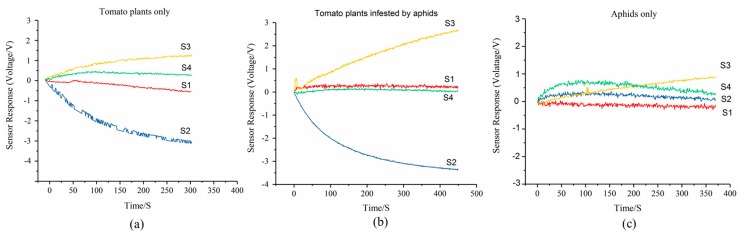
Sensor performance of the developed E-nose system for the healthy tomato plant (**a**), aphid-infested tomato plant (**b**), and aphids only (**c**) (sensor 1(S1), sensor 2(S2), sensor 3 (S3), and sensor 4 (S4)).

The E-nose system was developed to determine the health status of tomato plants by detecting VOCs released from these plants with and without aphid infestations. To verify its stability, the sensor sensitivity and reproducibility of the developed E-nose system were examined before detections. Figure 3a shows the sensor performance of MQ 138 under exposure of standard gases (alcohol, acetone, and methane liquid vapor) with different concentrations ranging from 10 mmol/L and 100 mmol/L, to 1000 mmol/L. The variation of voltage increased linearly with the growth of gas concentrations, demonstrating a reliable sensitivity towards target gases. Figure 3b shows the performance of the reproducibility test. Within three repeated tests towards headspace gas of healthy tomato plants, three successive curves with a response time of ~250 s and recovery time of ~50 s were observed, indicating a good reproducibility of the developed E-nose system. Given the sensor (MQ 138) performance towards the standard gases of methane vapor, it suggested that MQ 138 is probably a P-type. The sensor performance (averaged value) is shown in Figure 4, and it represents a typical sensor response. As exhibited, the sensor array displayed different patterns when exposed to healthy and aphid-stressed plants (Figure 4a,b, respectively). The aphid-stressed plants showed different sensor performances with much higher intensity than the healthy tomato plants, which indicated that the VOC profiles changed after the aphid infestation. In other words, tomato plants had a strong response to aphid attacks. It is interesting to note that the second sensor (S2) of the sensor array responded quickly and had an increasing sensor response with exposure to the infested tomato plant samples. That is because the second sensor (S2) was sensitive to organic volatile compounds (Table 1), especially terpenes, which are the major compound of VOCs from tomato plants, thereby, leading to an increasing sensor response. Comparing Figure 3a and Figure 4b, the sensor response of sensor 4 (S3), which was sensitive to alcohols, showed a fast and increasing sensor performance, indicating that some VOCs of alcohol class are being generated as a defense tool to combat aphid attack. The typical sensor performance of aphids only is shown in Figure 4c. S3 provided an increasing sensor performance but relative low intensity.

**Figure 5:**
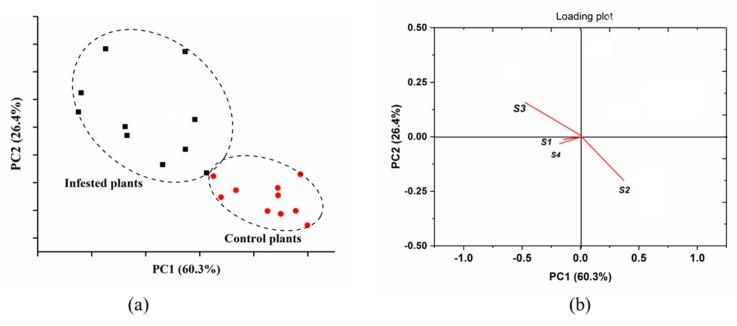
Principal Component Analysis (PCA) result (**a**) and loading plot (**b**) based on the E-nose data from healthy tomato plants and aphid-infested tomato plants.

**Figure 6:**
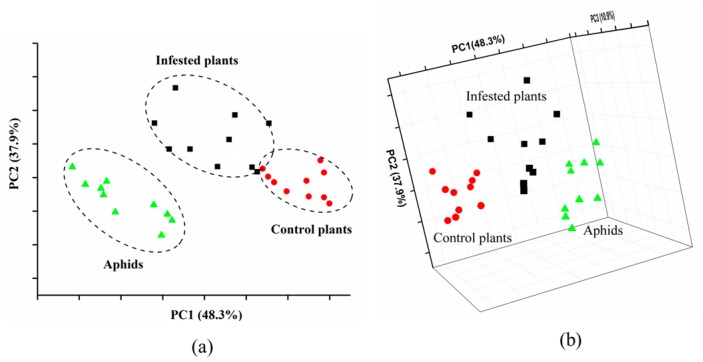
2D-PCA plot (**a**) and 3D-PCA plot (**b**) of healthy plants, aphid-infested plants, and aphids.

Given the satisfying sensor performance, which was based on the maximum value of each sensor response, the PCA was employed to classify aphid-stressed tomato plants from healthy plants at early stages. It should be noticed that the plants exposed to aphids for 2 days had no visible symptoms of an infestation. As shown in Figure 5, the PCA results provided the ability for classification between aphid-stressed and healthy plants, with the first two PCs accounting for 86.7% of the separations. Figure 6 shows a clear separation among groups of aphid-infested plants, healthy plants, and the aphids themselves through 2D and 3D PCA scatter plots. In Figure 5a, the aphid samples are located away from the other two nearby groups, while in the 3D plot of Figure 5b, all three groups are separated from each other, confirming the distinct difference of tomato plant VOC profiles before and after aphid attacks. The results indicated that the developed E-nose system was able to provide a rapid and non-invasive detection to distinguish aphid stressed-tomato plants from healthy ones at early stages of the infestation. Thus, the E-nose would have potential for insect control at early stages.

**Figure 7:**
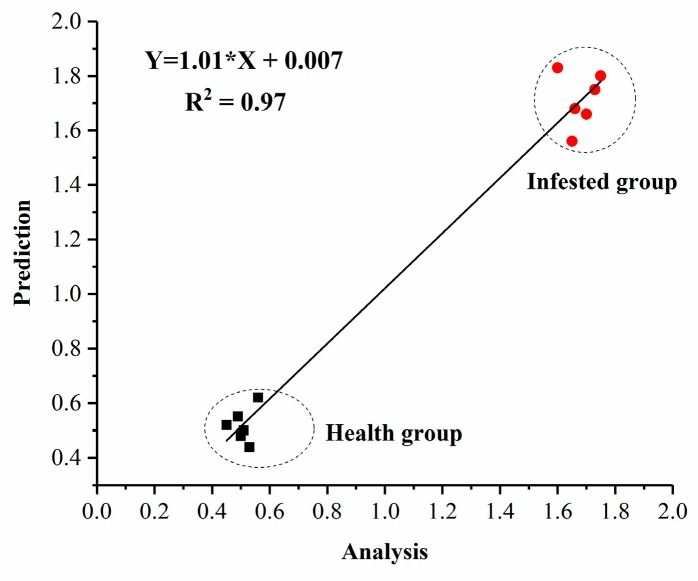
The regression curve of unknown vs known tomato plants based on sensors response.

In order to verify the efficiency of the developed E-nose system, unknown tomato plants were examined and compared with known ones that included healthy plants and infested plants (six duplicates for known samples and unknown samples, respectively). Results of the regression curve are shown in Figure 7. As indicated, all unknown plants were successfully classified into two different groups, and the high correlation (R^2^ = 0.97) of sensor responses (average of maximum value of sensor responses) between unknown and known plants indicated satisfactory reliability and efficiency.

### 3.2. Determination of VOCs 

In order to explore the hidden reasons that resulted in different sensor response patterns and to optimize the developed E-nose system to achieve higher accuracy and sensitivity, a full understanding of the changes in the types and concentrations of VOCs compounds was necessary. 

**Figure 8:**
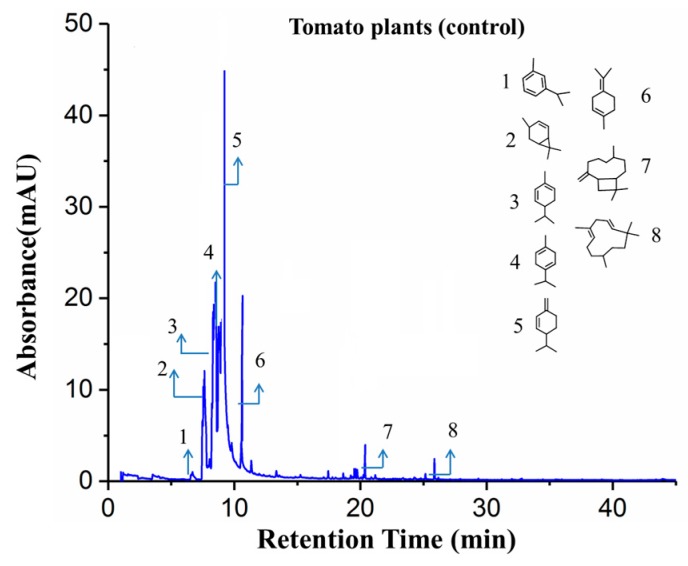
The GC-MS spectra of the VOCs released from healthy tomato plant (control).

**Figure 9:**
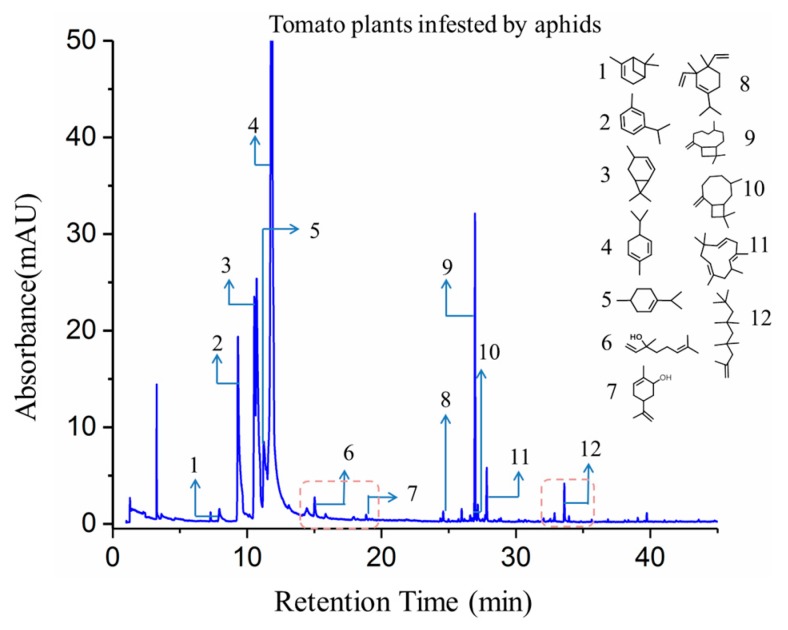
The GC-MS spectra of the VOCs released from aphid-stressed tomato plant.

**Figure 10:**
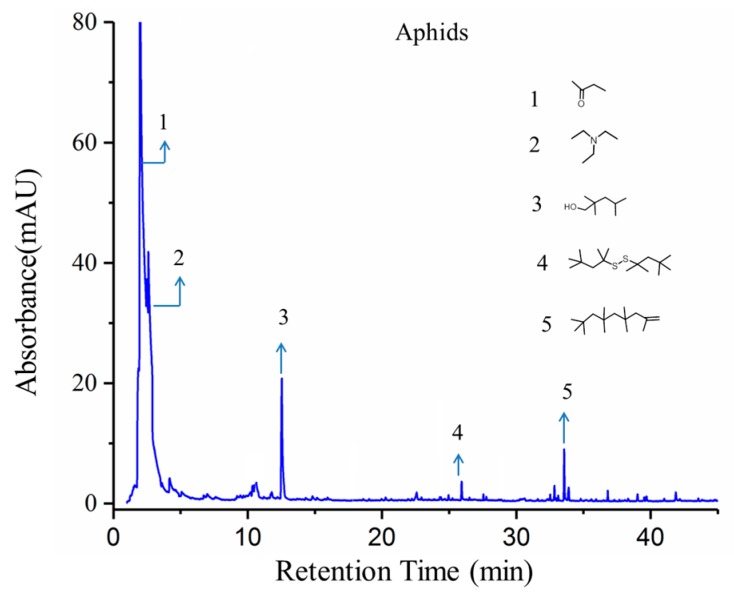
The GC-MS spectra of the VOCs of aphids.

Typical GC-MS spectrums of tested tomato plants with and without aphid infestation as well as the spectrum of aphids were shown in Figure 8, Figure 9 and Figure 10, with chemical structures of crucial volatile compounds (relative area% >1) identified. Variations between healthy tomato plants and aphid-stressed ones were obviously different. In comparison with healthy plants, two new volatile compounds (linalool and carveol) of the alcohol class and a sesquiterpene compound (2,4,4,6,6,8,8-heptamethyl-1-nonene) showed up, and they are marked by red rectangles in Figure 7. These two VOCs have been reported to have repellent or dispersal effects on aphids [29,30,31,32]. Specifically, the monoterpene alcohol linalool, although in trace concentration (3.52%), was shown to have a significant positive effect in response to aphid infestation [33,34]. Some research has reported that some components of natural essential oils, including linalool and carveol, have a high ovicidal activity towards aphid infestation [35,36,37,38]. Moreover, the concentration of caryophyllene significantly increased in infested plants, as shown in Figure 6. As a naturally producing sesquiterpene, caryophyllene can be endogenously released from plants, which are triggered by aphid attacks, to repulse aphids, and it plays a role in alarm activity [39]. 

For clarification, the corresponding VOCs of target GC-MS spectrums (Figure 8, Figure 9 and Figure 10) are listed in Table 3. It is notable that the relative concentration of *β*-phellandrene increased from 21.02% (un-infested) to 54.76% (infested) while the other sesquiterpene components, including *P*-cymene, *α*-phellandrene, *α*-terpinene, and limonene, were stable. That was probably because *β*-phellandrene was a compound that attracted and induced oviposition behavior in female hoverflies, which is a type of aphid of predators [40]. These findings suggest that, after being infested by aphids, tomato plants gave a strong response and defense to combat the attack by releasing new volatile compounds and altering the concentration of some terpenes compounds. These compounds are thought to play a role in attracting predators, or serving as an alarm activity or other communication between plants. Also, these findings are consistent with other reports [32,33,34,35,36,37,38,39,40]. The changes in VOC profiles of aphid-stressed plants compared to un-infested ones help to explain why the response of the second sensor S2, which is sensitive to terpenes, benzenes, and other organic compounds, was enhanced, but the response of the third sensor S3, which is sensitive to alcohols, had a positive rise in unstressed tomato plants. Comparing the lab-based GC-MS determination (Figure 8, Figure 9 and Figure 10) with the E-nose system tests (Figure 2b), it is worth noting that the response time of the E-nose system (6 min) was much less than that of the GC-MS test (50 mins), confirming the capabilities of the developed E-nose applying on pest diagnosis. 

Based on the GC-MS data, conclusions could be made that aphid-infested tomato plants emitted some new volatile compounds (such as terpene alcohols and terpenoids) as a defense against aphids; several specific terpenes might have played an info-chemical role at the tomato plant to insect interaction. More importantly, these VOC fingerprint changes explained behaviors of the sensors during the test and, thereby, provided a strong foundation and reliable tool for the insect diagnosis at early stages.

### 3.3. Seasonal Effects on the VOC Profiles of Tomato Plants 

**Figure 11:**
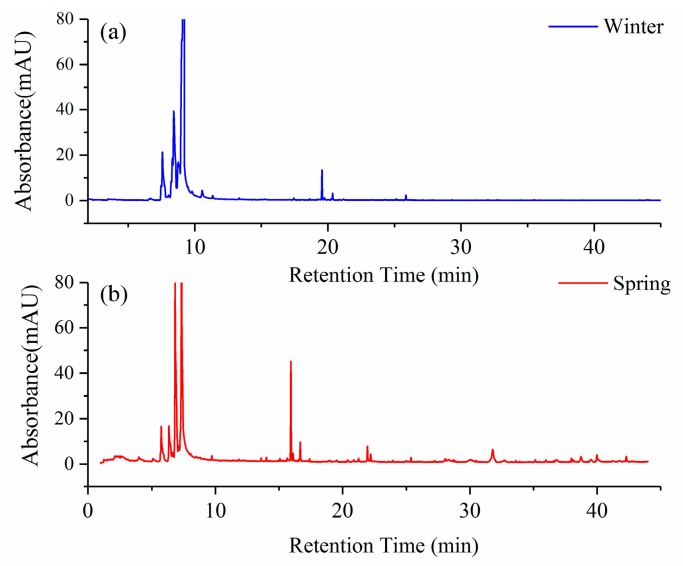
The GC-MS spectrum of tomato plants growing in spring and winter.

To certify the stability of the VOC profiles of tomato plants during the spring and winter growing seasons, plants were produced in a greenhouse in different seasons (spring and winter) with other conditions being the same. Headspace gases of selected plants were collected and analyzed using GC-MS, and the results of the VOCs profile are shown in Figure 11. The VOC profiles of tomato plants grown in winter and spring were found to be slightly different. Compared with the VOCs collected in winter, volatile compound intensity of 4-carene emitted from tomato plants in spring showed a slightly higher concentration, while the compound intensity of α-pinene and P-cymene were almost the same. Two terpene compounds of caryophyllene and humulene released from plants in spring were found to have a higher intensity than that released from plants in winter. Although some typical terpene compounds exhibited a higher intensity during spring growth, the VOC profile patterns were almost the same, guaranteeing the stability of E-nose performance and confirming the reliability of results. 

**Figure 12:**
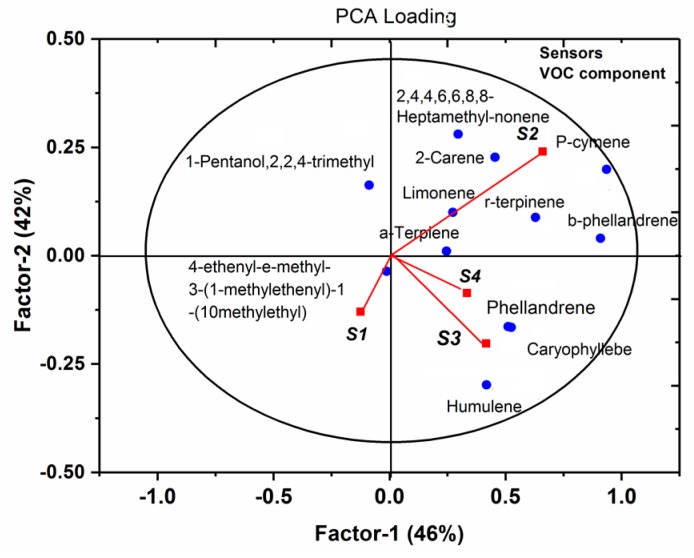
PCA loading plot between the sensors and VOCs compounds.

A relationship between sensor responses and VOC profiles was investigated using the PCA loading analysis. As shown in Figure 12, the second sensor (S2) and the third sensor (S3) had great weight coefficients on terpene compounds, confirming their sensitivity to terpenoids. The forth sensor shows a relatively lesser weight coefficient on ternoids compared with that of the former two sensors, while the sensor S1 had a negative reflection on either esters or terpenes, probably because of little sensor response from S1 when exposed to tomato plants. For further studies, the sensor array should be optimized by enhancing sensor sensitivity to esters and terpenes. Moreover, the performance of sensors and VOC profiles with and without aphid infestation agreed well with previous reports, indicating the reliability of the developed E-nose system [22,23,24,25,26,27]. 

## 4. Conclusions 

An E-nose system was successfully developed for a fast diagnosis of aphid-stressed tomato plants at early infestation stages. The GC-MS results indicated that aphid-stressed tomato plants released new volatile compounds (linalool, carveol, and nonane 2,2,4,4,6,8,8-heptamethyl) and enhanced some terpene compounds (e.g., caryopllyllene) for combating aphid attacks, and, thereby, were considered info-chemical biomarkers at the tomato plant-insect interaction. The changes of VOC fingerprints not only revealed the mechanism of different sensor responses towards different tomato plant samples, but also provided a baseline for further development and optimization of the E-nose system. The E-nose diagnosis results agreed well with the findings based on the GC-MS data. However, there were still some limitations in this study. For instance, clean air that carries gas for the rinsing gas chamber was not stable during the test, and the conditions (humidity and temperature) of the sampling system were not controlled. In further study, N2 will be used as carrier gas instead of using clean air, and humidity and temperature will be precisely controlled during sampling. Besides, more qualitative methods, such as artificial neutral network and K-nearest neighbor algorithm, will be employed for next qualitative evaluation. Further investigations are needed to validate the system reliability and repeatability before the E-nose can be practically used. 

## Figures and Tables

**Table 1:** Gases detected by each sensor employed in the E-nose system.

No.	Name	Sensed Gases
1	MQ 3(S1)	Alcohol
2	MQ 138 (S2)	Volatile compounds (aldehydes, alcohols, ketones and aromatic compounds)
3	TGS 2602 (S3)	Volatile organic compounds and odorous gases
4	MQ 135 (S4)	Benzene, Alcohol

**Table 2:** Operational parameter of the E-nose system test.

Items	Values
Sampling flow rate	100 mL/min
Rinse flow rate	100 mL/min
Temperature	22 °C
Relative humidity	45~48%
Carry gas for rinse	Clean air

**Table 3:** Typical VOCs released from aphid-infested tomato plants and healthy ones.

Peak Number	Peak Name	VOCs Identified						
		Tomato Plants Infested by Aphids		Tomato Plants (Healthy)		Aphids Only		Chemical Structure
		RT * (min)	Area (%)	RT * (min)	Area (%)	RT * (min)	Area (%)	
1	2-Butanone	--	--	--	--	1.847	63.75%	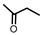
2	Triethylamine	--	--	--	--	2.46	24.99%	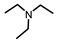
3	Alpha-pinene	7.27	0.1%	--	--	--	--	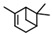
4	p-cymene	9.31	8.56%	7.47	8.03%	--	--	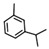
5	2-Carene	10.54	10.64%	8.22	7.37%	--	--	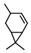
6	Limonene	11.23	4.02%	8.76	4.64%	--	--	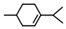
7	Alpha-phellandrene	10.70	13.92	8.42	14.18%			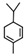
8	Gamma-terpinene	--	--	8.81	5.96%	--	--	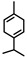
9	Beta-phellandrene	11.86	54.76	9.02	21.02	--	--	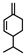
10	Cyclohexene, 1-methyl-4-(1-methylethylidenen)-	--	--	11.34	6.6%	--	--	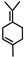
11	1-Pentanol, 2,2,4-trimethyl-	--	--	--	--	12.53	5.03%	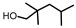
12	Linalool	15.85	3.52%	--	--	--	--	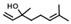
13	Carveol	18.85	1.04%	--	--	--	--	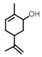
14	Cyclohexene, 4-ethenyl-4-methyl-3-(1-methylethenyl)-1-(1-methylethyl)-, (3R-trans)-	24.59	1.55%	--	--	--	--	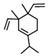
15	Caryophyllene	26.96	9..82%	20.35	1.2%	--	--	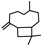
16	Humulene	27.83	0.96%	26.86	1.4%	--	--	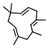
17	Disulfide, bis(1,1,3,3-tertramethylbutyl)	--	--	--	--	25.92	1.6%	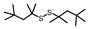
18	2,4,4,6,6,8,8-Heptamethyl-1-nonene	33.60	0.77%	--	--	32.83	0.66%	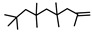

* RT: Retention time.
